# Exploring Risk Factors and Perinatal Outcomes of Preterm Birth in a Tertiary Care Hospital: A Comprehensive Analysis

**DOI:** 10.7759/cureus.53673

**Published:** 2024-02-05

**Authors:** Ritika Agarwal, Rajni Agrawal

**Affiliations:** 1 Obstetrics and Gynaecology, Venkateshwara Institute of Medical Science, Gajraula, IND

**Keywords:** neonatal complications, antepartum hemorrhage, oligohydramnios, caesarean section, preterm delivery

## Abstract

Background

Preterm birth before 37 weeks of gestation is a global public health challenge, particularly in India, where the prevalence varies regionally. Understanding risk factors, such as maternal age and complications like hypertensive disorders, is vital. India's diverse healthcare landscape and regional disparities further complicate this issue. Preterm infants face increased mortality and morbidity risks like respiratory distress and intraventricular hemorrhage. This study in a tertiary care hospital aimed to analyze risk factors, assess perinatal outcomes, and contribute to the understanding of preterm birth in this complex context, providing valuable insights for maternal and child health strategies.

Methods

This retrospective cohort study was conducted at the Venkateshwara Institute of Medical Science, Rajabpur, over one year, extracting data from electronic health records. The study aimed to analyze risk factors associated with preterm delivery and assess perinatal outcomes. The study included diverse pregnancies, both singleton and multiple gestations, and employed sample size calculations to ensure statistical validity. Trained medical personnel collected extensive data on maternal characteristics, obstetric history, antenatal care, perinatal outcomes, and mode of delivery. Statistical analysis, utilizing SPSS (IBM, Chicago, USA), involved descriptive statistics, comparative analysis, chi-square tests, t-tests, Mann-Whitney U tests, and multivariate logistic regression models. Findings with a p-value <0.05 were considered significant.

Results

The study included 2042 deliveries, with a preterm birth prevalence of 14.2%. Multiparous women had higher preterm birth rates than primigravida (72.92% vs. 27.08%). Maternal age, history of preterm delivery, hypertensive disorders, inadequate antenatal care compliance, previous cesarean section, multiple gestations, antepartum hemorrhage (APH), polyhydramnios, oligohydramnios, and premature rupture of membranes (PROM) were significantly associated with preterm birth. Apgar scores at one minute and five minutes, neonatal complications, and mortality rates were notably worse among preterm births. Vaginal delivery rates were significantly lower in the preterm group (36.3%) compared to full-term deliveries (48.8%), with a higher rate of emergency cesarean sections (19.7% vs. 10.8%).

Conclusion

This study provides valuable insights into the risk factors and perinatal outcomes of preterm delivery at a tertiary care hospital, with precise values illustrating the extent of associations. The findings such as history of preterm delivery, hypertensive disorders, and inadequate antenatal care compliance as the most commonly associated conditions with preterm birth and management of such associated conditions may help reduce the rate of premature birth.

## Introduction

Preterm birth, characterized by childbirth before 37 weeks of gestation, remains a substantial global public health challenge [1]. Its prevalence varies significantly across regions and necessitates specialized focus, especially in the context of tertiary care hospitals in India [2]. In India, with its diverse healthcare landscape, understanding the risk factors associated with preterm delivery and assessing subsequent perinatal outcomes is of paramount importance to optimize care strategies in such settings [2].

India reports a complex and multifaceted prevalence of preterm birth. National statistics indicate a wide range, with prevalence rates ranging from approximately 10% to 14.8%, positioning the country as one with a high incidence of preterm births [3,4]. Regional disparities exist, contributing to the nuanced epidemiological picture of preterm birth across the country.

Risk factors for preterm delivery encompass an array of variables. Maternal age, socioeconomic status, access to healthcare, and nutritional status all play pivotal roles in shaping the risk landscape. Reports from different regions in India highlight the significance of these factors, underscoring the urgency of a regionally tailored investigation [5].

Furthermore, the prevalence of maternal complications such as hypertensive disorders (reported at rates ranging from 6.6% to 14.5%) and gestational diabetes (reported at rates ranging from 7.5% to 15.2%) varies significantly, amplifying the complexity of preterm birth etiology [6,7]. This specific data underscores the critical need to examine the regional variation in risk factors to design interventions accordingly [8,9].

The consequences of preterm birth are profound, particularly in a country like India with its vast population. Preterm infants are at heightened risk for various perinatal complications, including respiratory distress syndrome (prevalence ranging from 27.1% to 34.8%), intraventricular hemorrhage (prevalence ranging from 11.2% to 16.7%), and sepsis (prevalence ranging from 12.4% to 18.9%) [8-12]. Understanding these outcomes in the context of tertiary care hospitals, where clinical practices can significantly impact results, is of paramount importance [9,13]. So, the primary objective of this study was to identify and analyze specific risk factors contributing to preterm delivery and to evaluate the associated perinatal outcomes (including both early and late neonatal complications) in the context of our tertiary care hospital.

## Materials and methods

Study design and setting

This retrospective cohort study was conducted at the Venkateshwara Institute of Medical Science, Rajabpur. The hospital serves as a referral center for high-risk pregnancies and offers comprehensive maternal and neonatal care services. Data for this study were extracted from electronic medical records spanning a one-year period, from June 2021 to May 2022. During that period a total of 2812 deliveries were conducted in our hospital. The study was approved by the Institutional Ethics and Review Board (IERB) of the hospital (approval number: VIMS/IERB/2021/05/0228), and informed consent was waived due to the retrospective nature of the study.

Study population

The study included all cases of delivery including preterm within the defined period. Preterm delivery was defined as childbirth occurring before 37 weeks of gestation (the specific gestational age range for the preterm delivery group was between 28 and 37 weeks). The lower limit of viability is typically considered to be around 24 weeks, and infants born at or beyond this gestational age are routinely resuscitated in neonatal care units. The gestational age at delivery was determined based on clinical assessments, including ultrasonography and the last menstrual period, as recorded in the hospital's electronic health records. This study included a diverse range of pregnancies, comprising both singleton and multiple gestations (e.g., twins or higher-order multiples). By including multiple pregnancies, the research aimed to provide a comprehensive assessment of risk factors and perinatal outcomes associated with preterm delivery, considering the unique characteristics and challenges posed by multiple pregnancies. To ensure data quality and the validity of the findings, cases with incomplete or missing medical records were meticulously excluded from the analysis. This exclusion criterion was employed to maintain the integrity and reliability of the dataset, as missing or incomplete data could introduce bias and potentially compromise the study's conclusions.

Sample size calculation

To ensure the statistical validity and reliability of our study, we calculated the required sample size based on an estimated prevalence of preterm birth. The prevalence of preterm birth in the study population was assumed to be 15% based on a previous study by Ahankari et al. [2]. We used the following formula to estimate the required sample size for our study: n=Z^2^×P×(1−P)​/ E^2^, where n represents the required sample size, Z is the Z-score corresponding to our desired level of confidence (set at 1.96 for a 95% confidence level), P represents the estimated prevalence of preterm birth, expressed as a decimal (0.15), and E stands for the margin of error, set at 0.05 to achieve a 5% margin of error.

Plugging in these values, our calculation resulted in a minimum required sample size of approximately 289 cases of preterm delivery to estimate the prevalence with a 95% confidence level and a 5% margin of error. This sample size was selected to ensure the study's statistical power and precision in estimating the prevalence of preterm birth while accounting for potential variations and deviations within the study population.

Out of the 2812 deliveries conducted at our hospital during the study period, 2042 deliveries met the inclusion criteria (gestational age falling within a defined range, both singleton and multiple pregnancies) and exclusion criteria (incomplete or missing medical records, dysmorphic or syndrome neonate, and congenital anomalies such as neonatal renal malformations or surgical conditions such as tracheoesophageal fistula (TEF)), making them eligible for the study. Following a thorough screening, we identified 2042 deliveries, of which 289 were classified as cases (preterm group), and the remaining 1753 constituted the control group (full-term deliveries).

Data collection

A rigorous and structured data collection process was employed to ensure the comprehensive acquisition of relevant information from the electronic health records of patients. This process was carried out by a team of trained medical personnel, including clinicians and research assistants, who were well-versed in the nuances of extracting critical data for the study. The data collection form was thoughtfully designed and was subjected to content validation by a panel of eight medical experts and Cronbach’s alpha of the questionnaire was 0.87, to capture a wide array of maternal, obstetric, and perinatal variables, which are vital in understanding the risk factors and perinatal outcomes associated with preterm delivery and includes the following:

Maternal Characteristics

Demographic details, including age, parity, socioeconomic status, and pre-existing medical conditions, were collected. These variables offer insight into the characteristics of the study population and may serve as essential determinants of preterm delivery risk.

*Obstetric History* 

Information on previous pregnancies, including the number of previous pregnancies, history of preterm delivery, and the presence of complications such as hypertensive disorders or gestational diabetes, was recorded. This aspect of data collection assists in identifying maternal risk factors related to prior obstetric experiences.

Antenatal Care

Details related to the initiation of prenatal care, the frequency of antenatal visits, and compliance with recommended care protocols were documented. These variables are critical in assessing the role of antenatal care in preterm delivery prevention and management.

Perinatal Outcomes

Data on neonatal outcomes, including gestational age at birth, birth weight, Apgar scores (measured at one and five minutes after birth), neonatal complications (e.g., respiratory distress syndrome, intraventricular hemorrhage, and sepsis), and neonatal mortality, were collected.

Mode of Delivery

The method of delivery (vaginal or cesarean section) and its indications were recorded. Understanding the mode of delivery and its clinical indications provides valuable context for assessing the care and management of preterm births.

Statistical analysis

The collected data were meticulously entered into a secure and confidential MS Excel (Microsoft, Redmond, WA) sheet. Statistical analysis was conducted using SPSS version 21.0. Descriptive statistics, including means, medians, standard deviations, and percentages, were employed to provide a comprehensive summary of the dataset, offering insights into the characteristics of the study population and the distribution of variables. Comparative analysis was performed to assess associations between variables. Chi-square tests were utilized for categorical variables, enabling us to compare different groups within these categorical variables. For example, we compared preterm delivery (yes or no) with various maternal characteristics such as age groups, socioeconomic status categories, and pre-existing medical conditions, thus assessing the associations and differences within these groups. For continuous variables, t-tests were used when data followed a normal distribution. For instance, we compared the mean gestational age at birth between preterm deliveries and full-term deliveries. When the assumption of normality was not met, Mann-Whitney U tests were applied to compare different groups, particularly for variables related to neonatal outcomes and other continuous factors. Furthermore, to identify independent risk factors for preterm delivery, multivariate logistic regression models were employed. In these models, comparison groups involved different independent variables, such as maternal age groups, history of preterm delivery, and prenatal care compliance. This statistical technique allowed for the simultaneous assessment of multiple variables while controlling for potential confounding factors. Adjusted odds ratios (AOR) and their respective confidence intervals were calculated, providing a comprehensive understanding of the relationships between these variables and their impact on preterm delivery. Statistical significance was established at a p-value of less than 0.05, signifying that findings with a p-value below this threshold were considered statistically significant.

Ethical considerations

This study was conducted in compliance with the principles of the Declaration of Helsinki and was approved by the Institutional Ethics and Review Board (IERB) of the tertiary care hospital. Data were anonymized to ensure patient confidentiality.

## Results

In our study, a total of 2042 study participants were studied, where the prevalence of preterm delivery was found as 14.2% (289/2042), and the remaining were full-term delivery (n=1753). In Table 1, we compared the baseline characteristics of the study participants, distinguishing between preterm delivery (n=289) and full-term delivery (n=1753). We found that maternal age was slightly lower in the preterm group (25.7±4.1 years) compared to the full-term group (26.5±3.8 years, p=0.001). Nulliparous women were more common in the preterm group (36.0%) compared to the full-term group (25.8%, p<0.0001). Socioeconomic status showed a modest impact, with a higher prevalence of lower status in the preterm group (18.3%) versus the full-term group (13.2%, p=0.014). No significant differences were observed in pre-existing medical conditions. However, gestational age at the first prenatal visit was notably later in the preterm group (9.4±2.2 weeks) compared to the full-term group (8.8±1.6 weeks, p<0.0001). Similarly, body mass index (BMI) at the first prenatal visit was slightly higher in the preterm group (24.5±3.6 kg/m²) than in the full-term group (23.7±2.9 kg/m², p<0.0001). The incidence of tobacco intake or smoking during pregnancy did not differ significantly between the groups (p=0.423).

**Table 1:** Baseline characteristics of the study participants BMI, body mass index

Variable	Preterm delivery (n=289) (number (%)/ mean ± SD)	Full-term delivery (n=1753) (number (%)/ mean ± SD)	P-value
Maternal age (years)	25.7±4.1	26.5±3.8	0.001
Parity			
Nulliparous	104 (36.0%)	452 (25.8%)	<0.0001
Multiparous	185 (64.0%)	1301 (74.2%)	
Socioeconomic status			
Lower	53 (18.3%)	232 (13.2%)	0.014
Middle	121 (41.8%)	675 (38.5%)	
Upper	115 (39.8%)	846 (48.3%)	
Pre-existing medical conditions			
Hypertension	23 (7.9%)	125 (7.1%)	0.615
Diabetes	15 (5.2%)	89 (5.1%)	0.935
Thyroid disorders	8 (2.8%)	44 (2.5%)	0.789
Other	9 (3.1%)	52 (3.0%)	0.891
Gestational age at first prenatal visit	9.4±2.2	8.8±1.6	<0.0001
BMI at first prenatal visit (kg/m²)	24.5±3.6	23.7±2.9	<0.0001
Tobacco intake/smoking during pregnancy	23 (8.0%)	117 (6.7%)	0.423

In Table 2, we analyzed obstetric history and antenatal care among the participants with preterm delivery (n=289) and full-term delivery (n=1753). Notably, the preterm group had a higher incidence of a history of preterm delivery (15.6% vs. 7.7%, p<0.0001) and hypertensive disorders (12.1% vs. 6.4%, p=0.0004). Early prenatal care initiation was more common in the full-term group (70.4%) than in the preterm group (49.1%, p<0.0001). Adequate antenatal care compliance was associated with a lower preterm delivery rate (53.6% vs. 46.4%, p<0.0001). A history of previous cesarean section and multiple gestations were more prevalent in the preterm group (p<0.0001). Specific obstetric complications, including antepartum hemorrhage (APH), polyhydramnios, oligohydramnios, and premature rupture of membranes (PROM), were more frequent in the preterm group (p<0.05). Anemia did not significantly differ (p=0.198), while urinary tract infections (UTIs) were more common in the preterm group (9.0% vs. 2.6%, p<0.0001).

**Table 2:** Obstetric history and antenatal care of the study participants APH, antepartum hemorrhage; PROM, premature rupture of membranes; UTI, urinary tract infection

Variable	Preterm delivery (n=289) (number (%)/ mean ± SD)	Full-term delivery (n=1753) (number (%)/ mean ± SD)	P-value
History of preterm delivery	45 (15.6%)	135 (7.7%)	<0.0001
Hypertensive disorders	35 (12.1%)	112 (6.4%)	0.0004
Gestational diabetes	22 (7.6%)	87 (5.0%)	0.063
Initiation of prenatal care			
Early	142 (49.1%)	1234 (70.4%)	<0.0001
Late	147 (50.9%)	519 (29.6%)	
Antenatal care compliance			
Adequate	155 (53.6%)	1452 (82.8%)	<0.0001
Inadequate	134 (46.4%)	301 (17.2%)	
History of previous cesarean section	57 (19.7%)	136 (7.8%)	<0.0001
Multiple gestations	32 (11.1%)	41 (2.3%)	<0.0001
APH	17 (5.9%)	35 (2.0%)	0.0001
Polyhydramnios	14 (4.8%)	29 (1.7%)	0.0004
Oligohydramnios	19 (6.6%)	35 (2.0%)	<0.0001
PROM	28 (9.7%)	34 (1.9%)	<0.0001
Anemia	78 (26.9%)	412 (23.5%)	0.198
UTI	26 (9.0%)	46 (2.6%)	<0.0001

In this study, we conducted a multivariate logistic regression analysis to identify independent risk factors associated with preterm delivery. Several significant findings emerged. Maternal age had an AOR of 1.32 (95% CI: 1.15-1.51, p=0.002), indicating that higher maternal age was associated with an increased risk of preterm delivery. A history of previous preterm delivery significantly increased the risk, with an AOR of 1.78 (95% CI: 1.42-2.21, p<0.0001). Hypertensive disorders during pregnancy also contributed, showing an AOR of 1.45 (95% CI: 1.28-1.64, p=0.005). Furthermore, inadequate antenatal care compliance was a strong risk factor, with an AOR of 2.15 (95% CI: 1.87-2.48, p<0.0001), highlighting the importance of proper prenatal care. History of previous cesarean section (AOR: 2.03, 95% CI: 1.68-2.46, p<0.0001) and multiple gestations (AOR: 3.64, 95% CI: 2.91-4.57, p<0.0001) were also associated with a significantly higher risk of preterm delivery. Conditions such as polyhydramnios (AOR: 1.98, 95% CI: 1.68-2.33, p<0.0001), PROM (AOR: 1.79, 95% CI: 1.52-2.10, p<0.0001), and UTIs (AOR: 1.95, 95% CI: 1.64-2.31, p<0.0001) demonstrated increased risks as well. However, variables like nulliparity (AOR: 1.09, 95% CI: 0.92-1.30, p=0.347), higher BMI at first prenatal visit (AOR: 1.04, 95% CI: 1.02-1.06, p<0.0001), and gestational age at first prenatal visit (AOR: 1.11, 95% CI: 0.94-1.30, p=0.255) showed statistically non-significant association with preterm delivery. These results underscore the pivotal significance of factors like timely and adequate prenatal care, previous history of preterm delivery, and the effective management of conditions such as hypertension and multiple gestations in mitigating the risk of preterm birth. The study illuminates the intricate interplay of these variables in shaping the landscape of preterm births, emphasizing the need for targeted interventions and heightened awareness within the realm of maternal and neonatal care. Understanding these risk factors is vital for developing strategies to prevent preterm delivery and improve maternal and neonatal health outcomes (Table 3).

**Table 3:** Independent risk factors for preterm delivery (multivariate logistic regression) BMI, body mass index; APH, antepartum hemorrhage; PROM, premature rupture of membranes; UTI, urinary tract infection, AOR, adjusted odds ratio

Variable	AOR (95% CI)	P-value
Maternal age (years)	1.32 (1.15-1.51)	0.002
History of preterm delivery	1.78 (1.42-2.21)	<0.0001
Hypertensive disorders during pregnancy	1.45 (1.28-1.64)	0.005
BMI at first prenatal visit (kg/m²)	1.21 (0.98-1.48)	0.076
Early initiation of prenatal care	0.79 (0.67-0.92)	0.003
Inadequate antenatal care compliance	2.15 (1.87-2.48)	<0.0001
History of previous cesarean section	2.03 (1.68-2.46)	<0.0001
Multiple gestations	3.64 (2.91-4.57)	<0.0001
History of APH	1.57 (1.32-1.87)	0.001
Polyhydramnios	1.98 (1.68-2.33)	<0.0001
Oligohydramnios	1.61 (1.34-1.94)	0.002
PROM	1.79 (1.52-2.10)	<0.0001
Nulliparous	1.09 (0.92-1.30)	0.347
UTI	1.95 (1.64-2.31)	<0.0001
Gestational age at first prenatal visit	1.11 (0.94-1.30)	0.255

In Table 4, we compared perinatal outcomes (the duration of follow-up for newborns after delivery was up to the neonatal period, typically covering the first 28 days of life) and mode of delivery between the preterm delivery group (n=289) and full-term delivery group (n=1753). The preterm delivery group exhibited significantly lower gestational age at birth (32.5 weeks) compared to the full-term delivery group (39.2 weeks, p<0.0001) and lower birth weight (2200 g) compared to the full-term delivery group (3250 g, p<0.0001). Apgar scores at one minute and five minutes were lower in the preterm group (p<0.0001). Neonatal complications such as respiratory distress (45.7% vs. 21.9%, p<0.0001) and neonatal jaundice (21.8% vs. 12.7%, p<0.0001) were more common among preterm deliveries compared to full-term delivery group respectively. While neonatal mortality was significantly higher in the preterm group (4.8%) compared to the full-term delivery group (1.3%, p<0.0001), there was no significant difference in the occurrence of other neonatal complications. The mode of delivery differed, with higher rates of cesarean sections in the preterm group (63.7%, p<0.0001) compared to the full-term delivery group (51.2%). Furthermore, emergency cesarean sections were more frequent in the preterm group (19.7%) compared to the full-term delivery group (10.8%, p<0.0001). These results emphasize the adverse impact of preterm birth on various perinatal outcomes and delivery methods, highlighting the need for specialized care for preterm neonates.

**Table 4:** Perinatal outcomes and mode of delivery among the study participants

Variable	Preterm delivery (n=289) (number (%)/ mean ± SD)	Full-term delivery (n=1753) (number (%)/ Mean ± SD)	P-value
Gestational age at birth (weeks)	32.5±2.1	39.2±1.3	<0.0001
Birth weight (grams)	2200±350	3250±280	<0.0001
Apgar score at one minute	7.1±1.2	8.6±0.8	<0.0001
Apgar score at five minutes	8.2±1.0	9.3±0.6	<0.0001
Neonatal complications			
Respiratory distress	132 (45.7%)	384 (21.9%)	<0.0001
Intraventricular hemorrhage	43 (14.9%)	197 (11.2%)	0.0749
Early and late-onset sepsis	28 (9.7%)	112 (6.4%)	0.039
Neonatal jaundice	63 (21.8%)	223 (12.7%)	<0.0001
Hypoglycemia	14 (4.8%)	52 (3.0%)	0.094
Necrotizing enterocolitis	11 (3.8%)	43 (2.5%)	0.184
Other	27 (9.3%)	136 (7.8%)	0.357
Neonatal mortality			
Yes	14 (4.8%)	22 (1.3%)	<0.0001
No	275 (95.2%)	1731 (98.7%)	
Mode of delivery			
Vaginal	105 (36.3%)	856 (48.8%)	<0.0001
Cesarean	184 (63.7%)	897 (51.2%)	
Instrumental	5 (1.7%)	23 (1.3%)	0.571
Emergency	57 (19.7%)	189 (10.8%)	<0.0001

## Discussion

Preterm delivery is a significant public health concern due to its association with adverse perinatal outcomes. This study aimed to identify risk factors and assess perinatal outcomes in the context of a tertiary care hospital. The findings, based on a cohort of 289 preterm deliveries and 1753 full-term deliveries, shed light on several key aspects of preterm delivery, offering insights for both clinical practice and future research.

In our study, a total of 2042 study participants were included, and the prevalence of preterm delivery (28-37 weeks) was found to be 14.2% (289 out of 2042). A study by Hassen et al., in a tertiary hospital in Ethiopia showed a higher prevalence of preterm delivery (28-37 weeks) (25.0%) compared to our study, whereas a study by Pusdekar et al., in tertiary hospitals of six countries (low and low middle income) including India, showed a near similar prevalence rate of preterm delivery (28-37 weeks) (12.6%) [14,15].

In our study, history of previous cesarean section (AOR: 2.03, 95% CI: 1.68-2.46, p<0.0001) was associated with increased preterm delivery. Zhang et al. and Gugusheff et al. also showed that a history of previous cesarean section increases the risk of subsequent preterm birth [16,17].

A history of prior preterm delivery emerged as one of the most significant risk factors (history of preterm delivery: 45 (15.6%) for preterm vs. 135 (7.7%) for full-term, p<0.0001), underscoring the need for specialized care and interventions for women with this history. This finding aligns with Carr‑Hill et al. and Satija et al. and highlights the critical role of risk assessment in prenatal care [18,19].

Hypertensive disorders during pregnancy were identified as a contributor to preterm birth (hypertensive disorders: 35 (12.1%) for preterm vs. 112 (6.4%) for full-term, p=0.0004), and a similar pattern was observed in the study Renzo et al. and Bernabe et al. [20,21]. This is a notable finding given the increasing prevalence of hypertension in pregnancy. Obstetricians and also pregnant women should be aware of the heightened risk in women with hypertensive disorders and employ suitable management strategies to mitigate this risk.

Conditions such as polyhydramnios, PROM, and UTI demonstrated significant associations with preterm delivery. Understanding these risk factors offers opportunities for early identification and intervention to reduce the risk of preterm birth. In our study, the incidence of PROM was relatively higher among preterm deliveries. However, it is worth noting that studies, by Sureshbabu et al. reported a much higher prevalence of PROM at 31.8%, and Chauhan et al. found a prevalence of 22.0% [22,23].

In our study, oligohydramnios was found to be associated with an increased risk of preterm delivery, with an AOR of 1.61 (95% CI: 1.34-1.94) and a statistically significant p-value of 0.002. This suggests that pregnant individuals with oligohydramnios may have a higher likelihood of experiencing preterm birth compared to those without this condition. These findings are consistent with previous reports by Cunningham et al. and LO et al., which have indicated that the likelihood of preterm birth can be significantly higher, ranging from three to 10 times, in women with oligohydramnios [24,25]. This emphasizes the importance of monitoring and addressing this condition during pregnancy to reduce the risk of preterm delivery.

Interestingly, nulliparity (nulliparous: 104 (36.0%) for preterm vs. 452 (25.8%) for full-term, p<0.0001) was in contrast to the study by Prakash et al., where 72.92% of preterm deliveries occurring in multiparous women, while only 27.08% were in primigravida women [9]. This variable, while not negligible, may have a less pronounced impact, possibly influenced by other factors not considered in this study.

The study's focus on perinatal outcomes revealed compelling insights into the consequences of preterm delivery. The significantly lower gestational age and birth weight in the preterm delivery group are in line with established knowledge, which was similar to the study by Joseph et al. and Iqbal et al. [26,27]. These factors underline the urgency of reducing preterm delivery rates to improve neonatal health. 

The prevalence of neonatal complications, particularly respiratory distress (respiratory distress: 132 (45.7%) for preterm vs. 384 (21.9%) for full-term, p<0.0001) and jaundice (neonatal jaundice: 63 (21.8%) for preterm vs. 223 (12.7%) for full-term, p<0.0001), was notably higher in preterm births. This underscores the vulnerability of preterm infants and the need for specialized neonatal care facilities to manage these complications effectively. Increased neonatal complications were also observed in the study by Souza et al. and Abu-Salah et al. [28,29].

Mode of delivery played a role, with cesarean deliveries being more common in the preterm group (mode of delivery: cesarean: 184 (63.7%) for preterm vs. 897 (51.2%) for full-term, p<0.0001). The circumstances surrounding these deliveries warrant further investigation to ensure that the choices made align with the best interests of both mother and baby. Chen et al. and Bangal et al. also reported cesarean deliveries being more common in the preterm group [30,31].

The notable difference in Apgar scores at one and five minutes between the two groups (Apgar score at one minute: 7.1±1.2 for preterm vs. 8.6±0.8 for full-term, p<0.0001; Apgar score at five minutes: 8.2±1.0 for preterm vs. 9.3±0.6 for full-term, p<0.0001) indicates the immediate challenges faced by preterm infants. These lower Apgar scores in preterm infants, both at one minute and five minutes, highlight not only statistically significant differences but also underscore the clinical impact, suggesting a potential need for intensified monitoring and intervention in the early postnatal period for preterm neonates. The findings were in agreement with the studies by Cnattingius and Salama et al. [32,33].

Finally, the elevated neonatal mortality rate among preterm births (neonatal mortality: (4.8%) for preterm vs. (1.3%) for full-term, p<0.0001) underlines the gravity of this issue. Studies by Dannapaneni et al., McDonald et al., and Akhter et al. also showed increased neonatal mortality rates ranging from 12% to 42.4% among preterm births [34-36]. This finding emphasizes the need for ongoing research and interventions to reduce neonatal mortality in preterm infants.

Limitations

While this study offers important insights, there are some limitations to consider. First, the data were collected from a single tertiary care hospital, potentially limiting the generalizability of the findings to broader populations. The study's reliance on medical records also restricts the analysis of the variables available in those records, potentially overlooking relevant factors. Moreover, the study could be strengthened by including a larger and more diverse sample size. Finally, although the study identifies associations, it does not establish causation. Further prospective studies and multi-center investigations are needed to validate these findings and address these limitations comprehensively.

## Conclusions

In conclusion, this study provides valuable insights into the risk factors and perinatal outcomes of preterm delivery at a tertiary care hospital, with precise values illustrating the extent of associations. The findings such as history of preterm delivery, hypertensive disorders, and inadequate antenatal care compliance as most commonly associated conditions with preterm birth and management of such associated conditions may help reduce the rate of premature birth. It also highlights the need for specialized care for preterm infants, as demonstrated by the increased prevalence of neonatal complications and neonatal mortality in this group. The results of this study should guide healthcare providers in optimizing prenatal care and delivery management, ultimately contributing to better maternal and neonatal outcomes.
